# Enhanced Field-Effect Control of Single-Layer WS_2_ Optical Features by hBN Full Encapsulation

**DOI:** 10.3390/nano12244425

**Published:** 2022-12-12

**Authors:** Anna Di Renzo, Onur Çakıroğlu, Felix Carrascoso, Hao Li, Giuseppe Gigli, Kenji Watanabe, Takashi Taniguchi, Carmen Munuera, Aurora Rizzo, Andres Castellanos-Gomez, Rosanna Mastria, Riccardo Frisenda

**Affiliations:** 1 Department of Mathematics and Physics “Ennio De Giorgi”, University of Salento, Via Arnesano, 73100 Lecce, Italy; 2 National Research Council, Institute of Nanotechnology (CNR-NANOTEC), Via Monteroni, 73100 Lecce, Italy; 3 Materials Science Factory, Instituto de Ciencia de Materiales de Madrid (ICMM-CSIC), E-28049 Madrid, Spain; 4 Research Center for Functional Materials, National Institute for Materials Science, 1-1 Namiki, Tsukuba 305-0044, Japan; 5 International Center for Materials Nanoarchitectonics, National Institute for Materials Science, 1-1 Namiki, Tsukuba 305-0044, Japan; 6 Physics Department, Sapienza University of Rome, Piazzale Aldo Moro 5, 00185 Rome, Italy

**Keywords:** van der Waals materials, WS_2_, hBN, photoluminescence, excitons

## Abstract

The field-effect control of the electrical and optical properties of two-dimensional (2D) van der Waals semiconductors (vdW) is one important aspect of this novel class of materials. Thanks to their reduced thickness and decreased screening, electric fields can easily penetrate in a 2D semiconductor and thus modulate their charge density and their properties. In literature, the field effect is routinely used to fabricate atomically thin field-effect transistors based on 2D semiconductors. Apart from the tuning of the electrical transport, it has been demonstrated that the field effect can also be used to modulate the excitonic optical emission of 2D transition metal dichalcogenides such as MoS_2_ or WSe_2_. In this paper, we present some recent experiments on the field-effect control of the optical and excitonic properties of the monolayer WS_2_. Using the deterministic transfer of van der Waals materials, we fabricate planar single-layer WS_2_ devices contacted by a gold electrode and partially sandwiched between two insulating hexagonal boron nitride (hBN) flakes. Thanks to the planar nature of the device, we can optically access both the hBN encapsulated and the unencapsulated WS_2_ regions and compare the field-effect control of the exciton population in the two cases. We find that the encapsulation strongly increases the range of tunability of the optical emission of WS_2_, allowing us to tune the photoluminescence emission from excitons-dominated to trions-dominated. We also discuss how the full encapsulation of WS_2_ with hBN helps reduce spurious hysteretic effects in the field-effect control of the optical properties, similar to what has been reported for 2D vdW field-effect transistors.

## 1. Introduction

In recent years, atomically thin semiconducting transition metal dichalcogenides (2D-TMDs) emerged as an appealing platform for the room-temperature implementation of excitonic systems. The reduced dimensionality, which characterises 2D-TMDs in the monolayer regime, results in a strong confinement of the charge carriers and reduced dielectric screening, inducing a strong Coulomb interaction with reported exciton binding energy up to 1 eV [1,2,3]. This allows for the room temperature optical response to be dominated by the physics of excitons [4]. These peculiarities have attracted a great deal of interest and are underpinning several appealing phenomena, including many-body states [5,6], spatially separated interlayer excitons in 2D heterostructures [7,8] and high-temperature exciton condensation in these systems [9]. The atomically thin nature of 2D-TMDs and their extreme sensitivity to the surrounding conditions also offer an unprecedented playground to influence and control exciton dynamics through external stimuli, such as strain engineering [10,11], the control of the dielectric environment [12] and the application of an electric field [13].

In the key challenge of manipulating the exciton physics of 2D-TMDs, electric-field control has been established as a promising and straightforward approach to modulate charge density in atomically thin materials and, thus, an effective tool to control both the electrical and the optical properties of 2D-TMDs. Indeed, due to the high excitons binding energies that allow for stable excitons at room temperature, the tuning of charge density in 2D-TMDs results in the generation of charged exciton bounded states apart from the neutral ones [14]. As a result, intrinsically *n*-doped 2D-TMDs exhibit not only the charge-neutral exciton feature (X_0_), but also a lower energy resonance corresponding to negative trions (X−) that consist of two electrons and one hole bound together through Coulomb interactions [14]. Apart from the intrinsic doping, these optical features can be further effectively modulated by electrical doping [15,16]. The effective tuning of the charge carrier’s population of 2D-TMDs through an electric field relies on the quality of the interface between the 2D-TMD monolayer and the gate dielectric oxide (usually SiO_2_) in a field-effect transistor (FET) device configuration. Indeed, the presence of trap states at the 2D-TMD/SiO_2_ interface strongly impacts the stability of the device operation and the overall device performances, giving rise to threshold voltage instability in FETs [17,18,19].

Herein, we have investigated the role of the hBN encapsulation in improving the gate-dependent optical properties of TMDs that are single-layer integrated in a single-electrode back-gated device, with a particular focus on the optical proprieties of WS_2_. Interestingly, we found that similarly to FETs, the field-effect control of the optical properties of the single-layer WS_2_ can also be strongly affected by traps, which introduce gate voltage hysteresis in the optical response and reduce the tunability of the excitons population. In this paper, we demonstrate that the full encapsulation of WS_2_ greatly enhances the optical tunability of the WS_2_ photoluminescence (PL) in response to an external electric-field provided by the back-gate, resulting in an improved modulation of the X_0_ and X− PL peaks intensity and in an increase in the energy separation between the X_0_ and X− PL peaks. In addition, we found that full encapsulation of the WS_2_ with insulating hexagonal boron nitride (hBN) also improves the field-effect control by reducing spurious hysteretic effects, with a beneficial effect on the tunability of the optical emission of WS_2_ thanks to the effective decoupling of the charge traps mediated by the hBN interlayer [20]. In general, hBN encapsulation is shown to lead to an enhancement of 2D WS_2_ optical quality by offering protection against unwanted doping contributions from substrates and chemicals, or physical adsorbates from the environment, resulting in cleaner spectra characterised by sharper emission from neutral and charged excitons [21].

## 2. Results and Discussions

**Figure 1:**
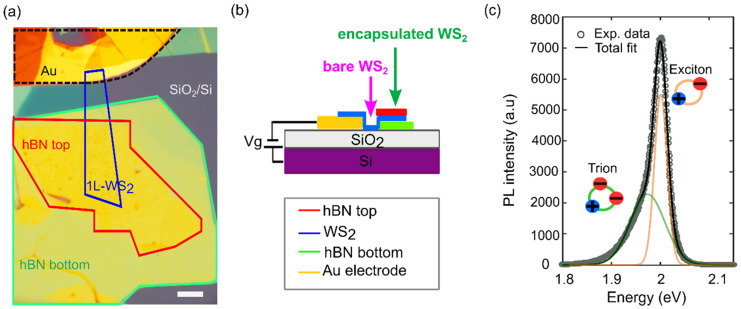
(**a**) Optical microscope images of the WS_2_-based device, and the scale bar corresponds to 20 μm. (**b**) Schematics of the system highlights to show the two different WS_2_ regions that can be experimentally accessed. (**c**) Photoluminescence spectrum of the hBN full encapsulated WS_2_ monolayer fitted to two peaks corresponding to the emission from neutral excitons (orange curve) and trions (green curve).

Figure 1a shows an optical microscopy picture of the fabricated device used to investigate the electric-field dependence of the exciton features of a bare and encapsulated WS_2_ single layer (1L-WS_2_). The WS_2_-based device consists of a partially encapsulated hBN/single-layer WS_2_/hBN heterostructure layered on top of a SiO_2_/Si substrate (SiO_2_ thickness 290 nm). We used a single gold electrode geometry in which the electrode is in direct contact with the WS_2_ monolayer, and the doping density can be varied by applying a voltage between the Si back-gate and the gold electrode (Figure 1b). The single-layer WS_2_ and hBN flakes were mechanically exfoliated (see Supplementary Materials, section ‘Sample Fabrication’) and sequentially transferred by an all-dry deterministic transfer procedure on the SiO_2_/Si substrate in contact with a pre-patterned Au electrode by using a polydimethylsiloxane stamp (Gel-Film WF × 4 6.0 mil by Gel-Pak) [22,23]. In particular, a bottom hBN flake (thickness~40 nm) was placed in close vicinity of the gold electrode and then the WS_2_ single layer was transferred, bridging the bottom hBN flake and the gold contact. Finally, the top hBN flake (thickness~20 nm) was positioned on top of the 1L-WS_2_/bottom hBN stack, covering only part of the WS_2_ flake (Figure 1a). This planar device geometry provides a great advantage to have, in the same device, two regions: the encapsulated (highlighted by the green arrow in Figure 1b) and the unencapsulated one (purple arrow in Figure 1b), both belonging to the same 1L-WS_2_ flake. This enables a direct understanding of the encapsulation effect and helps avoid possible WS_2_ flake-to-flake variations.

The monolayer thickness of the WS_2_ flake was confirmed through differential reflectance measurements before the heterostructure fabrication [24], as well as Raman spectroscopy (see Supplementary Materials, section ‘Raman characterization of the sample’ and Figure S1) and by PL spectroscopy of the WS_2_ monolayer. As expected, both the differential reflectance and the PL spectrum are characterised by a main feature at 2.01 eV (respectively, in the two cases, a dip and a peak) that is indicative of a single-layer WS_2_ flake [24,25]. Figure 1c shows the PL spectrum of the encapsulated WS_2_ recorded after the device fabrication, which is dominated by a prominent peak located at 2.001 eV, slightly red-shifted from the unencapsulated 1L-WS_2_, due to the change in the refractive index of the substrate from SiO_2_ to hBN [21]. With a closer inspection, one can see that the PL peak is skewed toward lower energies, showing a clear shoulder. In fact, the experimental data can be fitted to two Gaussian peaks centred, respectively, at 2.001 eV and 1.974 eV. These two features are excitonic in nature and can be assigned, respectively, to the recombination of excitons and trions at the K point in the band structure of the single-layer WS_2_ where the direct bandgap is located [25].

**Figure 2:**
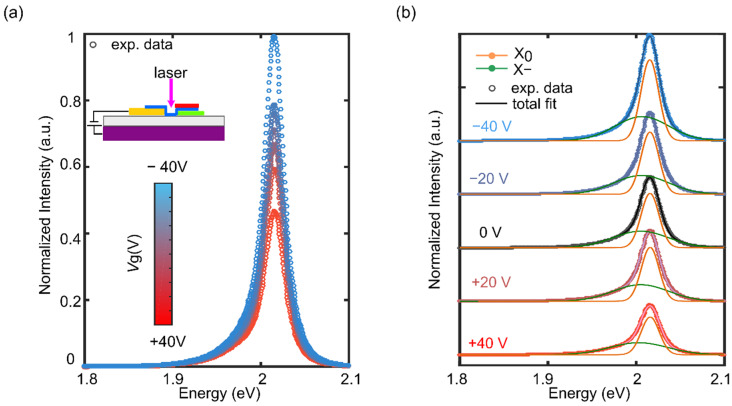
(**a**) Photoluminescence spectra of the 1L-WS_2_ unencapsulated region recorded at gate voltages between −40 V and 40 V. Inset: schematic of the probed region on the 1L-WS_2_-based device. (**b**) Photoluminescence spectra recorded at −40 V, −20 V, 0 V, 20 V and 40 V gate voltages fitted each to two peaks coming from exciton (orange curve) and trion (green curve) emission.

To investigate the electric-field modulation of the optical properties of the WS_2_ single layer and to reveal the influence of hBN encapsulation, we measured PL emission upon shining a focused laser beam on top of the bare WS_2_ or on the fully encapsulated regions, while sweeping the back-gate voltage (*V*_g_) between 40 V and −40 V. In this study, all the PL measurements were performed at room temperature using a 532 nm laser with a low excitation power of 4 μW, and a laser spot diameter of 1 μm. Figure 2a shows the recorded PL spectra of the bare 1L-WS_2_ acquired at different values of *V*_g_, between −40 V and 40 V. Similar to Figure 1c, the emission feature of the 1L-WS_2_ consists of a peak skewed toward lower energy ascribed to the radiative recombination of neutral excitons and negative trions. As can be observed from the plot, the total integrated PL intensity of the bare 1L-WS_2_ is weakly sensitive to the *V*_g_, being enhanced at the negative *V*_g_ and reduced at the positive *V*_g_. This behaviour is expected for a semiconductor characterised by an intrinsic *n*-doping as is the case of the 1L-WS_2_ [15,26]. The two-components fits of the 40 V, 20 V, 0 V, 20 V and −40 V PL spectra, presented in Figure 2b, show the influence of *V*_g_ on the X_0_ and X− peaks energies, and on the total integrated intensity two peaks [15,27]. In addition, we investigated the modulation of the 1L-WS_2_ excitonic properties by analysing the differential reflectance spectra as a function of the Vg (see Supplementary Materials, section ‘Gate dependent differential reflectance spectroscopy’).

**Figure 3:**
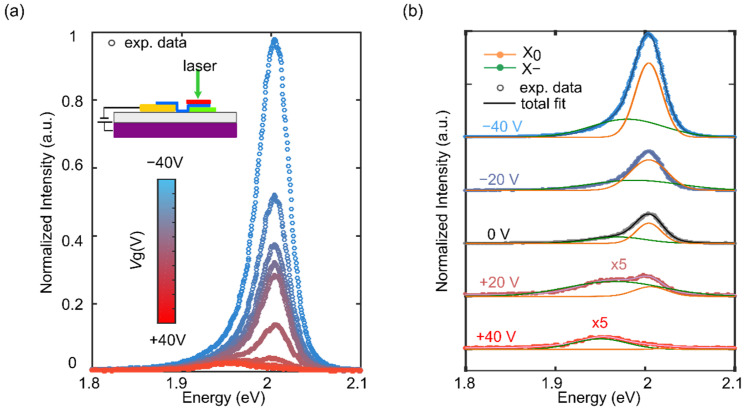
(**a**) Photoluminescence spectra of the 1L-WS_2_ hBN-encapsulated region recorded at gate voltages between −40 V and 40 V. Inset: schematic of the 1L-WS_2_ sample and of the probed region. (**b**) Photoluminescence spectra recorded at −40 V, −20 V, 0 V, 20 V and 40 V gate voltages fitted each to two peaks coming from exciton (orange curve) and trion (green curve) emission.

Differently from the bare 1L-WS_2_, the PL features of the hBN fully encapsulated WS_2_ single layer are more strongly affected from the electric-field, as shown in Figure 3a [20]. The total integrated intensity shows an abrupt decrease by sweeping the *V*_g_ from −40 V to 40 V, indicating the strong tunability of this parameter in the encapsulated case compared to the unencapsulated one. As can be seen in the fits of Figure 3b, at negative *V*_g_ the X_0_ peak is dominant, whereas at positive *V*_g_ it becomes almost undetectable due to the strong charge carrier injection given by the electrical doping [28,29]. In fact, at 40 V the X− peak remains the only prominent feature in the PL spectrum, thus showing an inversion of the excitonic population from excitons to trions. The shift of both the X_0_ and X− is also considerably enhanced thanks to the improved dielectric and traps environment provided by the hBN encapsulation, with a redshift of 30 meV of the X− peak and a blueshift of 16 meV of the X_0_ peak at *V*_g_ = 40 V [30,31].

**Figure 4:**
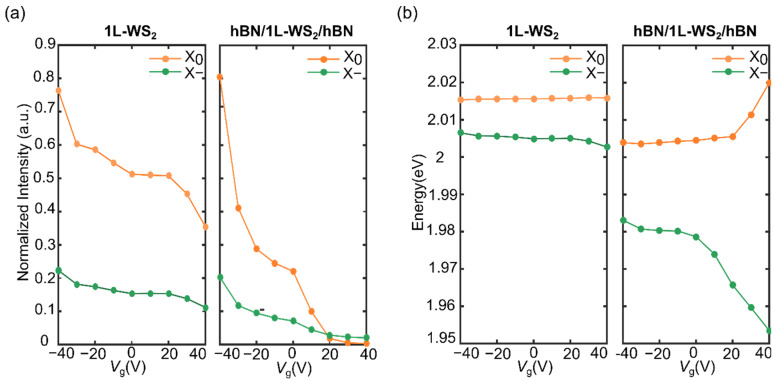
(**a**) Normalised integrated PL intensities of the X and X−peaks versus gate voltage on the 1LWS_2_ (left panel) and the hBN/WS_2_/hBN region (right panel). (**b**) Emission energy peaks of X and X− versus gate voltage on the 1L-WS_2_ (left panel) and the hBN/WS_2_/hBN region (right panel).

Figure 4 shows the results of the two-peaks fits of the PL spectra of the bare 1L-WS_2_ and the hBN fully encapsulated 1L-WS2 as a function of *V*_g_. The intensity of the peaks is reported in panel a and the centre of the peaks in panel b. The peaks intensity shows, in both the unencapsulated and encapsulated cases, a decrease when going toward positive gate voltages, but this decrease is shallower in the first case and more abrupt in the second case. Focusing on the neutral exciton, we can observe that its intensity is reduced by 50% in the unencapsulated WS_2_ by sweeping the voltage from negative to positive. Nevertheless, the X_0_ peak is always more intense than the X− peak in the full voltage range. On the other hand, the X_0_ peak intensity of the neutral exciton is reduced by 99% in the encapsulated sample. Moreover, the encapsulated case shows an inversion of the dominant peak for voltages larger than 20 V, indicating that for these voltages, the generation and recombination of trions is favoured over the neutral excitons.

The energies of the peaks in the two 1L-WS_2_ regions also show analogous behaviour, with the encapsulated case showing a much stronger dependence on *V*_g_ than the unencapsulated one, in which the peak’s energies appear almost constant. The sweep of *V*_g_ from −40 V to 40 V induces, in the encapsulated case, a redshift of almost 30 meV for the X-peak position and a slight blueshift 16 meV for the X_0_ peak. In addition, the energy splitting between the X_0_ and X− remarkably increases in the encapsulated case when sweeping *V*_g_ from −40 V to 40 V, resulting in an energy separation that goes from 20 meV to 66 meV, which in this latter case is about five times higher than in the bare 1L-WS_2_. Interestingly, the full encapsulation enables improved field-effect control considering that, in previous reports that exploited the electric-field control of the optical properties of the bare WS_2_, a maximum splitting of 34 meV had been achieved only by using very high operational voltages [27,31]. It is worth noting that both the X_0_ and X− peaks are initially redshifted because of the encapsulation, as previously reported [32]. In the literature, the energy splitting between the X and X− has been defined as the dissociation energy of trions, which, in the case of constant trion binding energy, is mainly dependent on the Fermi-level position [16]. The energy splitting thus increases when increasing the electron doping, as a consequence of the rising of the Fermi level (see Figure 4b) [15,33]. On the other hand, the blueshift of the exciton energy is ascribed to a reduction in the exciton binding energy resulting from electron doping [16,34]. Thus, the enhanced energy splitting between the X_0_ and X− reported in the case of the fully hBN encapsulated WS_2_ represents clear evidence of the more effective electrical doping of the 1L-WS_2_ and the enhanced field-effect control enabled by the hBN full encapsulation.

**Figure 5:**
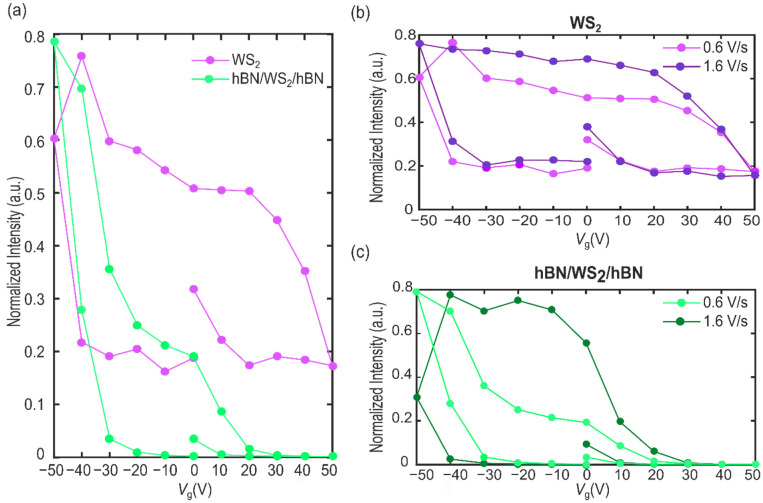
(**a**) Comparison of the gate-dependent optical hysteresis of the X_0_ peak integrated intensity in 1L-WS_2_ (magenta curve) and hBN/1L-WS_2_/hBN (green curve). (**b**,**c**) Comparison of the hysteresis with different back-gate voltage sweep rates for the X_0_ intensity in 1L-WS_2_ (**b**) and in hBN/WS_2_/hBN (**c**) at 0.6 V/s and at 1.6 V/s.

To gain insight into the field-effect control of the optical properties of the single-layer WS_2_ and to further investigate the advantage of the hBN encapsulation, we also performed a full gate sweep measurement, which consists of forward and reverse sweeps, first from 0 V to 50 V, then from 50 V to −50 V and finally sweeping back from −50 V to 0 V, with a gate voltage changing rate of 0.6 V/s, and focusing on the emission of the neutral exciton X_0_. Figure 5a shows the intensity of the X_0_ peaks in the two 1L-WS_2_ when performing this full gate sweep. As can be seen, the curves do not overlap in the forward and backward *V*_g_ sweep. Nevertheless, this non-ideal hysteretic behaviour is remarkably reduced when considering the PL emission of the hBN/WS_2_/hBN region. In detail, the encapsulation by few-layers hBN induces a 52% reduction in the hysteresis thanks to the effective decoupling of the charge traps and the atmosphere adsorbates from the 1L-WS_2_. However, even with hBN encapsulation, the hysteresis is not completely suppressed. This could be due to the presence of residual impurities (i.e., PDMS residues and/or water molecules) unintentionally introduced during the fabrication process, or to defects and traps intrinsic to the WS_2_ crystal [20].

Finally, we have investigated the optical hysteresis at different *V*_g_ sweep rates. Figure 5b,c show the neutral exciton PL peak intensity recorded from the bare 1L-WS_2_ and hBN/WS_2_/hBN obtained applying two different back-gate sweep rates, namely 0.6 V/s and 1.6 V/s, for the full gate sweep measurement. We observe the overall trend of reduced hysteresis as the sweep rate decreases; however, the behaviour is different in the bare and the fully encapsulated 1L-WS_2_ regions. While the bare 1L-WS2 exhibits a hysteresis that is almost not influenced by the speed, showing only a slight reduction estimated in 19% of the hysteresis voltage window, in the hBN/WS_2_/hBN the hysteresis at 0.6 V/s sweep rate is significantly lower if compared to the 1.6 V/s rate, showing a reduction of 59%. This suggests that, in the latter case, the traps present in the system are more short-lived and less detrimental for the overall optical tunability and field-effect control.

## 3. Conclusions

In conclusion, we demonstrated the tuning of excitonic emission in the monolayer WS_2_ exploiting an externally applied electric field in a single-electrode device. We considered both the bare and hBN fully encapsulated WS_2_ single layer and we found that the hBN encapsulation greatly enhances the optical tunability in response to the electric field. In detail, the PL response of the full hBN encapsulated WS_2_ monolayer changes dramatically, resulting in an increase of the energy splitting between neutral excitons and trions from 20 meV at −40 V up to 66 meV for voltages of 40 V, as the Fermi level rises. In addition, the enhanced effect of doping enabled by the full encapsulation allows us to fully suppress the neutral exciton generation and recombination at positive voltages, resulting in a PL spectrum completely dominated by the trions emission. The hBN encapsulation is also effective at reducing the hysteresis observed in the voltage-dependent PL, providing adequate protection from the trap states that can be present, for example, at the interface between SiO_2_ and WS_2_. These findings demonstrate that field-effect control combined with hBN encapsulation represents a practical approach to control and/or enhance the radiative excitonic emission in single-layer WS_2_ at room temperature toward improved exciton-based optoelectronic applications.

## Data Availability

The data presented in this study are in the paper and/or the Supplemental Information. Additional data related to this paper may be requested from R.F. (riccardo.frisenda@uniroma1.it).

## Supplementary Materials

The following supporting information can be downloaded at: https://www.mdpi.com/article/10.3390/nano12244425/s1: Sample Fabrication, Raman characterization of the sample, Gate dependent differential reflectance spectroscopy. References [35,36,37,38,39,40,41] are cited in the supplementary materials.

## References

[1] Qiu D.Y., da Jornada F.H., Louie S. (2013). G Optical Spectrum of MoS2: Many-Body Effects and Diversity of Exciton. Phys. Rev. Lett..

[2] Cheiwchanchamnangij T., Lambrecht W.R.L. (2012). Quasiparticle Band Structure Calculation of Monolayer, Bilayer, and Bulk MoS2. Phys. Rev. B Condens. Matter Mater. Phys..

[3] Ramasubramaniam A.A. (2012). Large Excitonic Effects in Monolayers of Molybdenum and Tungsten Dichalcogenides. Phys. Rev. B Condens. Matter Mater. Phys..

[4] Mueller T., Malic E. (2018). Exciton Physics and Device Application of Two-Dimensional Transition Metal Dichalcogenide Semiconductors. Npj 2D Mater. Appl..

[5] Van Tuan D., Scharf B., Wang Z., Shan J., Mak K.F., Žutić I., Dery H. (2019). Probing Many-Body Interactions in Monolayer Transition-Metal Dichalcogenides. Phys. Rev. B.

[6] Xiao K., Yan T., Liu Q., Yang S., Kan C., Duan R., Liu Z., Cui X. (2021). Many-Body Effect on Optical Properties of Monolayer Molybdenum Diselenide. J. Phys. Chem. Lett..

[7] Rivera P., Schaibley J.R., Jones A.M., Ross J.S., Wu S., Aivazian G., Klement P., Seyler K., Clark G., Ghimire N.J. (2015). Observation of Long-Lived Interlayer Excitons in Monolayer MoSe2-WSe2 Heterostructures. Nat. Commun..

[8] Chiu M.H., Li M.Y., Zhang W., Hsu W.T., Chang W.H., Terrones M., Terrones H., Li L.J. (2014). Spectroscopic Signatures for Interlayer Coupling in MoS2-WSe2 van Der Waals Stacking. ACS Nano.

[9] Wang Z., Rhodes D.A., Watanabe K., Taniguchi T., Hone J.C., Shan J., Mak K.F. (2019). Evidence of High-Temperature Exciton Condensation in Two-Dimensional Atomic Double Layers. Nature.

[10] Carrascoso F., Li H., Frisenda R., Castellanos-Gomez A. (2021). Strain Engineering in Single-, Bi- and Tri-Layer MoS2, MoSe2, WS2 and WSe2. Nano Res..

[11] Carrascoso F., Frisenda R., Castellanos-Gomez A. (2022). Biaxial versus Uniaxial Strain Tuning of Single-Layer MoS2. Nano Mater. Sci..

[12] Peimyoo N., Wu H.Y., Escolar J., de Sanctis A., Prando G., Vollmer F., Withers F., Riis-Jensen A.C., Craciun M.F., Thygesen K.S. (2020). Engineering Dielectric Screening for Potential-Well Arrays of Excitons in 2D Materials. ACS Appl. Mater. Interfaces.

[13] Radisavljevic B., Radenovic A., Brivio J., Giacometti V., Kis A.A. (2011). Single-Layer MoS2 Transistors. Nat. Nanotechnol..

[14] Ross J.S., Wu S., Yu H., Ghimire N.J., Jones A.M., Aivazian G., Yan J., Mandrus D.G., Xiao D., Yao W. (2013). Electrical Control of Neutral and Charged Excitons in a Monolayer Semiconductor. Nat. Commun..

[15] Mak K.F., He K., Lee C., Lee G.H., Hone J., Heinz T.F., Shan J. (2013). Tightly Bound Trions in Monolayer MoS2. Nat. Mater..

[16] Shang J., Shen X., Cong C., Peimyoo N., Cao B., Eginligil M., Yu T. (2015). Observation of excitonic fine structure in a 2D transition-metal dichalcogenide semiconductor. ACS Nano.

[17] Egginger M., Bauer S., Schwödiauer R., Neugebauer H., Sariciftci N.S. (2009). Current versus gate voltage hysteresis in organic field effect transistors. Mon. Fur Chem..

[18] Ghatak S., Pal A.N., Ghosh A. (2011). Nature of Electronic States in Atomically Thin MoS2 Field-Effect Transistors. ACS Nano.

[19] Guo Y., Wei X., Shu J., Liu B., Yin J., Guan C., Han Y., Gao S., Chen Q. (2015). Charge Trapping at the MoS2-SiO_2_ Interface and Its Effects on the Characteristics of MoS2 Metal-Oxide-Semiconductor Field Effect Transistors. Appl. Phys. Lett..

[20] Lee C., Rathi S., Khan M.A., Lim D., Kim Y., Yun S.J., Youn D.H., Watanabe K., Taniguchi T., Kim G.H. (2018). Comparison of Trapped Charges and Hysteresis Behavior in HBN Encapsulated Single MoS2 Flake Based Field Effect Transistors on SiO_2_ and HBN Substrates. Nanotechnology.

[21] Wierzbowski J., Klein J., Sigger F., Straubinger C., Kremser M., Taniguchi T., Watanabe K., Wurstbauer U., Holleitner A.W., Kaniber M. (2017). Direct Exciton Emission from Atomically Thin Transition Metal Dichalcogenide Heterostructures Near the Lifetime Limit. Sci. Rep..

[22] Castellanos-Gomez A., Buscema M., Molenaar R., Singh V., Janssen L., van der Zant H.S.J., Steele G.A. (2014). Deterministic Transfer of Two-Dimensional Materials by All-Dry Viscoelastic Stamping. 2D Mater..

[23] Zhao Q., Wang T., Ryu Y.K., Frisenda R., Castellanos-Gomez A.J. (2020). An Inexpensive System for the Deterministic Transfer of 2D Materials. J. Phys. Mater..

[24] Niu Y., Gonzalez-Abad S., Frisenda R., Marauhn P., Drüppel M., Gant P., Schmidt R., Taghavi N.S., Barcons D., Molina-Mendoza A.J. (2018). Thickness-Dependent Differential Reflectance Spectra of Monolayer and Few-Layer MoS2, MoSe2, WS2 and WSe2. Nanomaterials.

[25] Cong C., Shang J., Wang Y., Yu T. (2018). Optical Properties of 2D Semiconductor WS2. Adv. Opt. Mater..

[26] Ayari A., Cobas E., Ogundadegbe O., Fuhrer M.S. (2007). Realization and Electrical Characterization of Ultrathin Crystals of Layered Transition-Metal Dichalcogenides. J. Appl. Phys..

[27] Plechinger G., Nagler P., Kraus J., Paradiso N., Strunk C., Schüller C., Korn T. (2015). Identification of excitons, trions and biexcitons in single-layer WS2. Phys. Status Solidi Rapid Res. Lett..

[28] Illarionov Y.Y., Knobloch T., Jech M., Lanza M., Akinwande D., Vexler M.I., Mueller T., Lemme M.C., Fiori G., Schwierz F. (2020). Insulators for 2D nanoelectronics: The gap to bridge. Nat. Commun..

[29] Illarionov Y.Y., Rzepa G., Waltl M., Knobloch T., Grill A., Furchi M.M., Mueller T., Grasser T. (2016). The role of charge trapping in MoS2/SiO2 and MoS2/hBN field-effect transistors. 2D Mater..

[30] Cavalcante L.S.R., da Costa D.R., Farias G.A., Reichman D.R., Chaves A. (2018). Stark Shift of Excitons and Trions in Two-Dimensional Materials. Phys. Rev. B.

[31] Zhu B., Chen X., Cui X. (2015). Exciton Binding Energy of Monolayer WS2. Sci. Rep..

[32] Sun Z., Beaumariage J., Xu K., Liang J., Hou S., Forrest S.R., Fullerton-Shirey S.K., Snoke D.W. (2019). Electric-Field-Induced Optical Hysteresis in Single-Layer WSe2. Appl. Phys. Lett..

[33] Huard V., Cox R.T., Saminadayar K., Arnoult A., Tatarenko S. (2000). Bound states in optical absorption of semiconductor quantum wells containing a two-dimensional electron Gas. Phys. Rev. Lett..

[34] Zhang C., Wang H., Chan W., Manolatou C., Rana F. (2014). bsorption of Light by Excitons and Trions in Monolayers of Metal Dichalcogenide Mo S2: Experiments and Theory. Phys. Rev. B Condens. Matter Mater. Phys..

[35] Taghavi N.S., Gant P., Huang P., Niehues I., Schmidt R., de Vasconcellos S.M., Bratschitsch R., García-Hernández M., Frisenda R., Castellanos-Gomez A. (2019). Thickness Determination of MoS2, MoSe2, WS2 and WSe2 on Transparent Stamps Used for Deterministic Transfer of 2D Materials. Nano Res..

[36] Taniguchi T., Watanabe K.J. (2007). Synthesis of high-purity boron nitride single crystals under high pressure by using Ba-Bn solvent. Cryst. Growth.

[37] Berkdemir A., Gutiérrez H.R., Botello-Méndez A.R., Perea-López N., Elías A.L., Chia C.I., Wang B., Crespi V.H., López-Urías F., Charlier J.C. (2013). Identification of individual and few layers of WS2 using Raman Spectroscopy. Sci. Rep..

[38] Castellanos-Gomez A., Quereda J., van der Meulen H.P., Agraït N., Rubio-Bollinger G. (2016). Spatially Resolved Optical Absorption Spectroscopy of Single- and Few-Layer MoS2 by Hyperspectral Imaging. Nanotechnology.

[39] Splendiani A., Sun L., Zhang Y., Li T., Kim J., Chim C.Y., Galli G., Wang F. (2010). Emerging Photoluminescence in Monolayer MoS2. Nano Lett..

[40] Zhao W., Ghorannevis Z., Chu L., Toh M., Kloc C., Tan P.H., Eda G. (2013). Evolution of Electronic Structure in Atomically Thin- Sheets of Ws 2 and Wse2. ACS Nano.

[41] Shree S., Paradisanos I., Marie X., Robert C., Urbaszek B. (2021). Guide to Optical Spectroscopy of Layered Semiconductors. Nat. Rev. Phys..

