# Supplementary material for: Enhanced Field-Effect Control of Single-Layer WS2 Optical Features by hBN Full Encapsulation
Source: Nanomaterials (Basel). 2022 Dec 12;12(24):4425. doi: 10.3390/nano12244425 (PMC9788429; doi:10.3390/nano12244425)
Supplement: Supplementary file 1 [file nanomaterials-12-04425-s001.zip › nanomaterials-2065122-supplementary.pdf]

Supporting Information

Enhanced Field-Effect Control of Single-Layer WS_2 Optical Features by hBN Full Encapsulation

Anna Di Renzo [1,2], Onur Çakıroğlu [3], Felix Carrascoso [3], Hao Li [3], Giuseppe Gigli [1,2], Kenji Watanabe [4], Takashi Taniguchi [5], Carmen Munuera [3], Aurora Rizzo [2], Andres Castellanos-Gomez [3,*], Rosanna Mastria [2,*] and Riccardo Frisenda [3,6,*]

1 Department of Mathematics and Physics "Ennio De Giorgi", University of Salento, Via Arnesano, 73100 Lecce, Italy; anna.direnzo@unisalento.it (A.D.R.); giuseppe.gigli@unisalento.it (G.G.)
2 National Research Council, Institute of Nanotechnology (CNR-NANOTEC), Via Monteroni, 73100 Lecce, Italy; aurora.rizzo@nanotec.cnr.it
3 Materials Science Factory, Instituto de Ciencia de Materiales de Madrid (ICMM-CSIC), E-28049 Madrid, Spain; onur.cakiroglu@csic.es (O.Ç.); felix.c@csic.es (F.C.); lihao-zzu@outlook.com (H.L.); cmunuera@icmm.csic.es (C.M.)
4 Research Center for Functional Materials, National Institute for Materials Science, 1-1 Namiki,
Tsukuba 305-0044, Japan; watanabe.kenji.aml@nims.go.jp
5 International Center for Materials Nanoarchitectonics, National Institute for Materials Science, 1-1 Namiki, Tsukuba 305-0044, Japan; taniguchi.takashi@nims.go.jp
6 Physics Department, Sapienza University of Rome, Piazzale Aldo Moro 5, 00185 Rome, Italy
* Correspondence: andres.castellanos@csic.es (A.C.-G.), rosanna.mastria@nanotec.cnr.it (R.M.); riccardo.frisenda@uniroma1.it (R.F.)

Sample fabrication

Single-layer WS_2 and few-layer hBN flakes were mechanically exfoliated from bulk crystals with Nitto SPV 224 tape and transferred onto the surface of a Gel-Film (Gel-Pak WF ×4 6.0 mil) substrate. Then single-layer flakes of WS_2 were identified on the PDMS stamp with an optical microscope operated in transmission mode[1] and their thickness confirmed by differential reflectance.

Synthetic bulk WS_2 crystals were purchased from HQ Graphene.

Bulk hBN crystals were grown by K. Watanabe and T. Taniguchi.[2]

Raman characterization of the sample

Figure S1 shows the Raman signals coming from single-layer WS_2 (between 200 cm^{-1} and 500 cm^{-1}) and hBN (between 1200 cm^{-1} and 1500 cm^{-1}). The top panels have been recorded on bare WS_2 and bare hBN regions while the bottom panels have been measured in the hBN/WS_2/hBN stack region. The spectra collected on the WS_2 monolayer show typical Raman feature for this 2D material. [3] For laser excitation of 532 nm, the Raman spectrum is dominated by the 2LA mode at ~ 352 cm^{-1}, E^1_{2g} at ~356 cm^{-1} and A_{1g} at ~ 418 cm^{-1}.

Regarding hBN, the Raman spectrum is dominated by E_{2g} peak at 1362 cm^{-1}.

Gate dependent differential reflectance spectroscopy:

In order to verify the tunability of the optical properties by means of the electric-field, preliminary gate-dependent differential reflectance measurements were carried out on the devices.

For this measurements, the device is mounted under the objective of an optical microscope (Motic BA310 Met-T) system supplemented with a homebuilt micro-reflectance module based on a fiber- coupled CCD spectrometer (CCS200/M, Thorlabs).[4] The differential reflectance spectrum was calculated as (R-R0)/R, where R is the intensity reflected by the flake, R0 the intensity reflected by the substrate.[5] The absorbance spectra show two main peak features at 2.02 eV and 2.4 eV, respectively associated to direct band gap transitions at the K point of the Brillouin zone that generates excitons labelled A and B,[6] but Figure S2 focuses mainly on the characteristics of exciton A, which is also prominent in photoluminescence.[7]

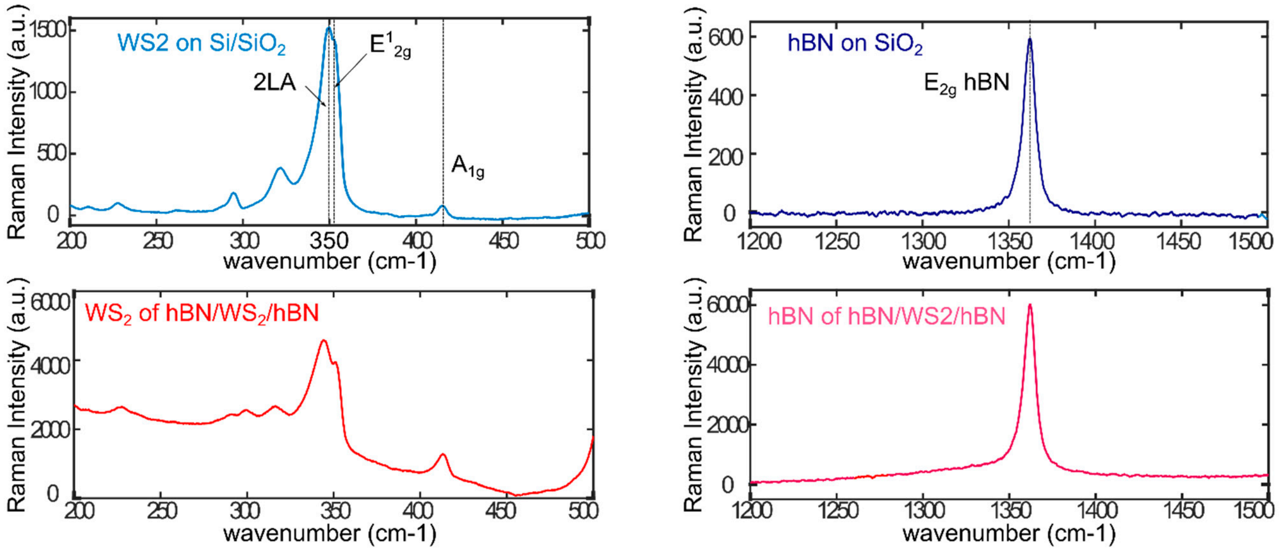

Figure S1. Room-temperature Raman spectra from the unencapsulated monolayer WS_2 region (top left), from the bottom hBN (top right) and from the hBN/WS_2/hBN stack region (bottom left and bottom right), using a 532 nm laser excitation. The main peaks due to WS_2 and hBN have been labeled.

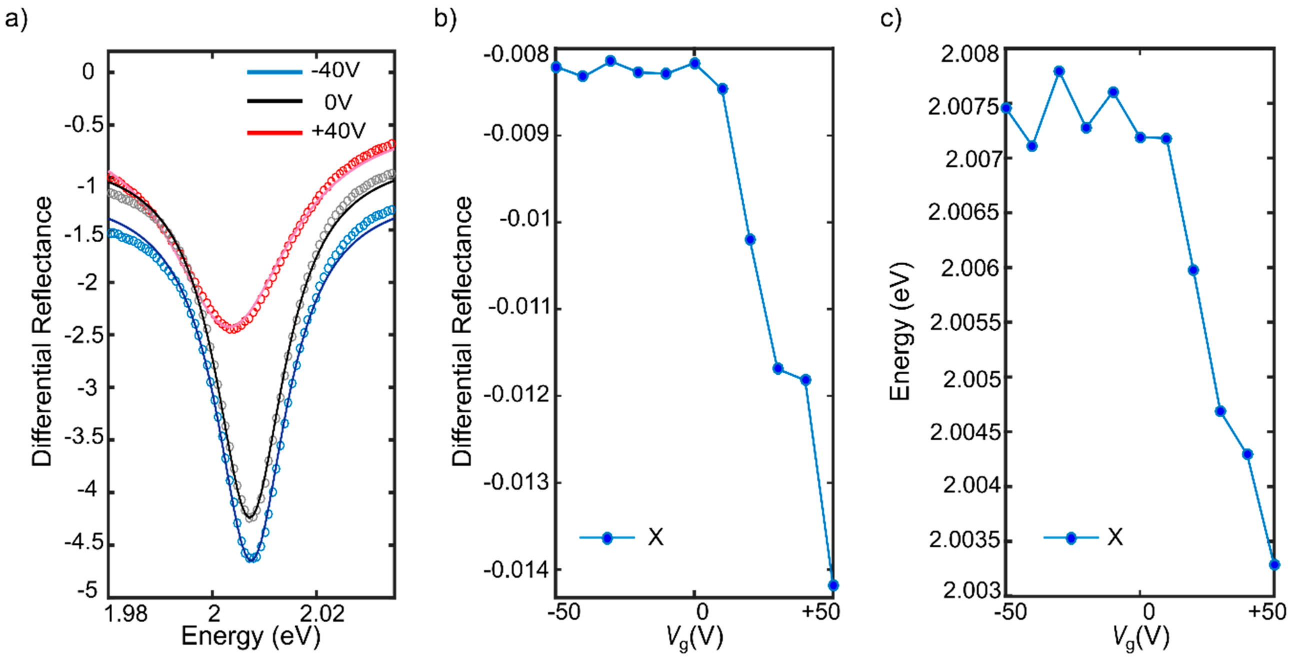

Figure S2. (a) Differential Reflectance spectra of the 1L-WS_2 sample at the back-gate voltages between -40V and 40 V. (b) Differential reflectance intensity of the A peak versus gate voltage. (c) Gate-dependent optical hysteresis the for A peak differential reflectance intensity in 1L-WS_2 FET.

Figure S2a shows the differential reflectance spectra of WS_2 FET under gate voltages, namely -40 V, 0 V and 40 V. We observed an overall reduction of the optical absorbance in correspondance of the A peak by increasing electron doping. Moreover, this effect is also accompanied by a red shift of the main peak. Fig S2b shows, the intensity variation of the peak as a function of voltage ranging from -40 V and 40 V with a step voltage of 10 V. We also observed the hysteresis behaviour in the differential reflectance performing a full gate sweep from -50 V to 50 V (Figure S2c). It is worth to note that was not possible to appreciate a reflectance signal on the WS_2 single-layer encapsulated with hBN, due to thin film destruvtive interference related to the thicknesses of hBN and SiO_2 that strongly influence the visibility of exciton resonances in absorption measurements.[8]

References

[1] N.S. Taghavi, P. Gant, P. Huang, I. Niehues, R. Schmidt, S. Michaelis de Vasconcellos, R. Bratschitsch, M. García-Hernández, R. Frisenda, and A. Castellanos-Gomez, Nano Research **12**, 1691 (2019).

[2] T.Taniguchi & K. Watanabe J. Cryst. Growth **303**, 525 (2007).

[3] Berkdemir, A.; Gutiérrez, H.R.; Botello-Méndez, A.R.; Perea-López, N.; Elías, A.L.; Chia, C.I.; Wang, B.; Crespi, V.H.; López-Urías, F.; Charlier, J.C.; et al.. Sci Rep 2013, **3**, doi:10.1038/srep01755.

[4] Y. Niu, S. Gonzalez-Abad, R. Frisenda, P. Marauhn, M. Drüppel, P. Gant, R. Schmidt, N.S. Taghavi, D. Barcons, A.J. Molina-Mendoza, S.M. de Vasconcellos, R. Bratschitsch, D.P. de Lara, M. Rohlfing, and A. Castellanos-Gomez, Nanomaterials **8**, 725(2018).

[5] A. Castellanos-Gomez, J. Quereda, H.P. van der Meulen, N. Agraït, and G. Rubio-Bollinger, Nanotechnology **27**, 115705(2016).

[6] A. Splendiani, L. Sun, Y. Zhang, T. Li, J. Kim, C.Y. Chim, G. Galli, and F. Wang, Nano Letters **10**, 1271 (2010).

[7] W. Zhao, Z. Ghorannevis, L. Chu, M. Toh, C. Kloc, P.H. Tan, and G. Eda, ACS Nano **7**, 791 (2013).

[8] S. Shree, I. Paradisanos, X. Marie, C. Robert, and B. Urbaszek, Nature Reviews Physics **3**, 39 (2021).
